# Transcriptional reprogramming of gene expression in bovine somatic cell chromatin transfer embryos

**DOI:** 10.1186/1471-2164-10-190

**Published:** 2009-04-24

**Authors:** Nelida Rodriguez-Osorio, Zhongde Wang, Poothappillai Kasinathan, Grier P Page, James M Robl, Erdogan Memili

**Affiliations:** 1 Department of Animal and Dairy Sciences, Mississippi State University, Starkville, MS, USA; 2 Hematech Inc, Sioux Falls, SD, USA; 3 University of Alabama-Birmingham, Birmingham AL, USA; 4 Grupo CENTAURO, Universidad de Antioquia, Medellín, Antioquía, Colombia

## Abstract

**Background:**

Successful reprogramming of a somatic genome to produce a healthy clone by somatic cells nuclear transfer (SCNT) is a rare event and the mechanisms involved in this process are poorly defined. When serial or successive rounds of cloning are performed, blastocyst and full term development rates decline even further with the increasing rounds of cloning. Identifying the "cumulative errors" could reveal the epigenetic reprogramming blocks in animal cloning.

**Results:**

Bovine clones from up to four generations of successive cloning were produced by chromatin transfer (CT). Using Affymetrix bovine microarrays we determined that the transcriptomes of blastocysts derived from the first and the fourth rounds of cloning (CT1 and CT4 respectively) have undergone an extensive reprogramming and were more similar to blastocysts derived from *in vitro *fertilization (IVF) than to the donor cells used for the first and the fourth rounds of chromatin transfer (DC1 and DC4 respectively). However a set of transcripts in the cloned embryos showed a misregulated pattern when compared to IVF embryos. Among the genes consistently upregulated in both CT groups compared to the IVF embryos were genes involved in regulation of cytoskeleton and cell shape. Among the genes consistently upregulated in IVF embryos compared to both CT groups were genes involved in chromatin remodelling and stress coping.

**Conclusion:**

The present study provides a data set that could contribute in our understanding of epigenetic errors in somatic cell chromatin transfer. Identifying "cumulative errors" after serial cloning could reveal some of the epigenetic reprogramming blocks shedding light on the reprogramming process, important for both basic and applied research.

## Background

The process of early embryonic development is determined by activation of the embryonic genome, which for bovine embryos begins as a "minor genome activation" at the 1-cell stage [1] ascending to a "major genome activation" during the 8-cell to 16-cell stage [2]. In the absence of proper genome activation, the developing embryo will die because it can no longer support its essential developmental functions [3,4]. In the case of embryos produced by somatic cell nuclear transfer (SCNT) the somatic nucleus has to be reprogrammed in order to restart and continue the developmental process. It is believed that, guided by the ooplasm, the somatic nucleus aborts its own program of somatic gene expression and re-establishes a particular program of embryonic gene expression necessary for normal embryo development [4].

Embryos produced by SCNT have lower developmental rates than their *in vitro *and *in vivo *produced counterparts [5]. Embryos produced by SCNT also have a greater incidence of apoptosis and consequently a lower number of cells [6]. Additionally, SCNT derived embryos have greater rates of embryo and fetal mortality, stillbirths and perinatal deaths, which bring down the overall efficiency of cloning. These alterations may be caused, at least partially, by incomplete epigenetic reprogramming of the somatic nuclei [5,7]. Somatic cell chromatin transfer (SCCT) attempts to facilitate the reprogramming process by exposing the somatic cells, prior to the transfer, to a mitotic cell extract, which is supposed to induce chromosome condensation and promote the removal and solubilisation of nuclear factors, enhancing nuclear remodelling [8]. Compared with nuclear transfer, SCCT shows greater survival of cloned calves up to at least 1 month and could be a useful tool in understanding the mechanisms of reprogramming [8]. Remarkably, a recent study did not detect any significant differences in the global gene expression profiles of SCCT and SCNT embryos [9].

Embryos derived from nuclear transfer have an abnormal pattern of DNA methylation, in some cases resembling that of somatic cells [10-12]. This aberrant DNA methylation pattern has been inversely correlated with the developmental potential of the cloned embryos [13]. Treatment of donor cells with DNA demethylation agents, prior the nuclear transfer, may remove epigenetic marks improving the ability of the somatic cells to be fully reprogrammed by the recipient karyoplast [14]. Global alteration of gene expression has been another finding in embryos produced by cloning. The abnormal expression of genes playing important roles in early embryonic development, implantation and fetal development is of particular interest. Conversely, other studies have reported a significant reprogramming for SCNT embryos by the blastocyst stage and similar transcriptome profiles to those of embryos produced *in vitro *or *in vivo*, suggesting that defects in gene expression for SCNT embryos may occur later during redifferentiation and organogenesis [15,16].

Among the abnormally expressed genes reported in bovine cloned embryos are IL6, FGF4, and FGFr2 [17]; FGF4, DNMT1, Mash2, HSP70, and interferon tau [18]; Acrogranin, Cdx2, and ERR2 [19]. Cytokeratin 19, Cytokeratin 8, Vimentin, Hsp27, Nidogen2 and MHC-I [20]; HDAC-1, 2, and 3, DNMT3A, and OCT4 [21]. Lower levels of transcripts involved in the retinoic acid signalling pathway (RARB, CRAB1, HLA-A, THBS2, and SERPINB5) were reported for cloned bovine embryos [22]. There have been conflicting results when it comes to the expression of particular genes in SCNT and IVF embryos. Such is the case of the developmentally important POU5F1 gene, which has been reported as misregulated in cloned embryos compared to IVF derived blastocysts in some studies [21,23], while being detected at similar concentration in others [17,24].

SCNT is often used for the production of human proteins in the milk of transgenic animals. For the achievement of some specific transgenic phenotypes, multiple genetic modifications need to be completed through sequential modifications in primary cells prior to nuclear transfer [25]. Since transfection and selection of transgenic cells requires nearly the entire lifespan of a cell, only one genetic modification can be completed in each cell lifespan [26]. Therefore, consecutive rounds of cloning (also referred to "repeated cloning", "serial cloning", "recloning" or "nuclear recycling") are performed. It has been proposed that consecutive rounds of cloning, allow for rejuvenation and selection of transformed cultured cells [27-30] and that it may improve the efficiency of SCNT by increasing the reprogramming potential of the somatic cells [31,32]. Conversely, other reports suggest that epigenetic errors could accumulate in the embryos as a result of serial cloning and prolonged *in vitro *culture decreasing cloning efficiency. After serial cloning up to the sixth generation was performed in mice, no signs of telomere shortening or premature ageing were observed. However, cloning efficiency significantly decreased with increasing rounds of cloning [33]. A greatly reduced *in vitro *and *in vivo *developmental capacity was reported for bovine embryos derived after several rounds of serial cloning [34,35]. It has been suggested that extended culture associated with transfection and selection procedures may induce changes of somatic cells, which decrease the efficiency of nuclear transfer and that these changes cannot be reversed by recloning [36].

The objective of the present study was to identify the "cumulative errors" on global gene expression, caused by serial rounds of chromatin transfer, by comparing the transcriptome profile of IVF derived blastocysts to that of SCCT derived blastocysts from the first and fourth rounds of cloning (CT1 and CT4) using oligonucleotide microarray analysis (Affymetrix Bovine GeneChips). Donor cells used for first and fourth rounds of cloning (DC1 and DC4) were also the target of the study as we compared the global gene expression of the SCCT embryos with their respective donor cells. Additionally, we analyzed the expression patterns of a panel of selected genes, in fetal fibroblasts obtained from foetuses from zero to fifth rounds of chromatin transfer. Our results show that a substantial reprogramming has taken place in the cloned embryos from both generations of chromatin transfer. However, there was a set of differential expressed genes in both groups of cloned embryos compared to their IVF counterparts. The number and functions of these genes could suggest accumulative misregulations probably caused by the successive rounds of cloning.

## Results

### Isolation of RNA

**Figure 1:**
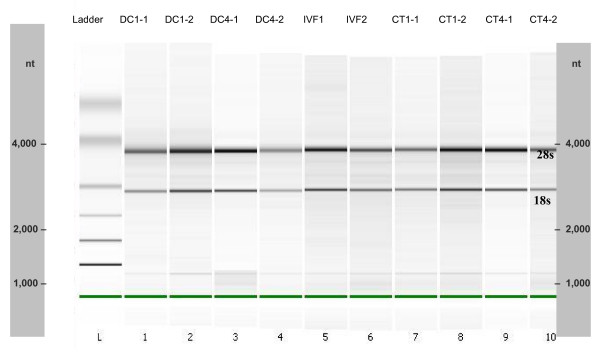
**Agilent bioanalyzer gel-like image of total RNA**. The image shows a total RNA gel like-image produced by the Bioanalyzer. (Ten out of the 15 samples used in the microarray experiment are shown since no more than 11 samples can be run at one time). Lane L: Size markers. Lanes 1 and 2: total RNA from 10^6 ^donor cells used for the first round of embryonic cloning. Lanes 3 and 4: total RNA from 10^6 ^donor cell used for the fourth round of cloning. Lanes 5 and 6: total RNA from a pool of 3 In Vitro Produced embryos. Lanes 7 and 8: total RNA from a pool of 3 embryos produced by the first round of chromatin transfer. Lanes 9 and 10: total RNA from a pool of 3 embryos produced by the fourth round of chromatin transfer. The 28S and 18s distinctive ribosomal RNA bands are observed for all samples.

On average 12.2 ng of total RNA were isolated from pools of 3 embryos (between 3.2 and 4.5 ng per blastocyst). The RNA integrity ranged from 1.8 to 1.96, based on the ratio between the 28S and 18S ribosomal RNA bands from the Bioanalyzer gel-like image (Figure 1).

### Transcriptome analyses

The Affymetrix GeneChip^® ^Bovine Genome Array contains 24,129 probe sets representing over 23,998 bovine transcripts, including assemblies from approximately 19,000 UniGene Clusters. In order to assess the influence of the two cycles of linear amplification, on the representation of original transcripts, we compared microarray experiments from one-cycle and two-cycle amplifications using total RNA from DC1. The results showed that amplification of messages using 1 *vs*. 2 cycles were highly consistent with a correlation coefficient of 0.93 (data not shown). These data confirm the manufacturer's results using 1 and 2 cycles of linear amplification.

Microarray experiments were performed in three biological replicates for all blastocysts (CT1, CT4 and IVF) and donor cells (DC1 and DC4). Images were processed with GCOS and data extracted using MAS 5.0. However, one of the CT1 blastocyst chips did not pass the quality control analysis [37] and was excluded from the study. The analyses for CT1 are based on the remaining two chips in this group, which showed an appropriate p-value distribution. The GCOS software expression data report showed that 56% of the probe sets were called "Present" (P) for all donor cell chips. This number was lower for all blastocyst chips with 44%, 41%, and 47% for IVF, CT1, and CT4 respectively. Probe sets that were called "Absent" (A) in all the samples were excluded from the analysis. Therefore only 16,521 probe sets were included in the analysis.

**Figure 2:**
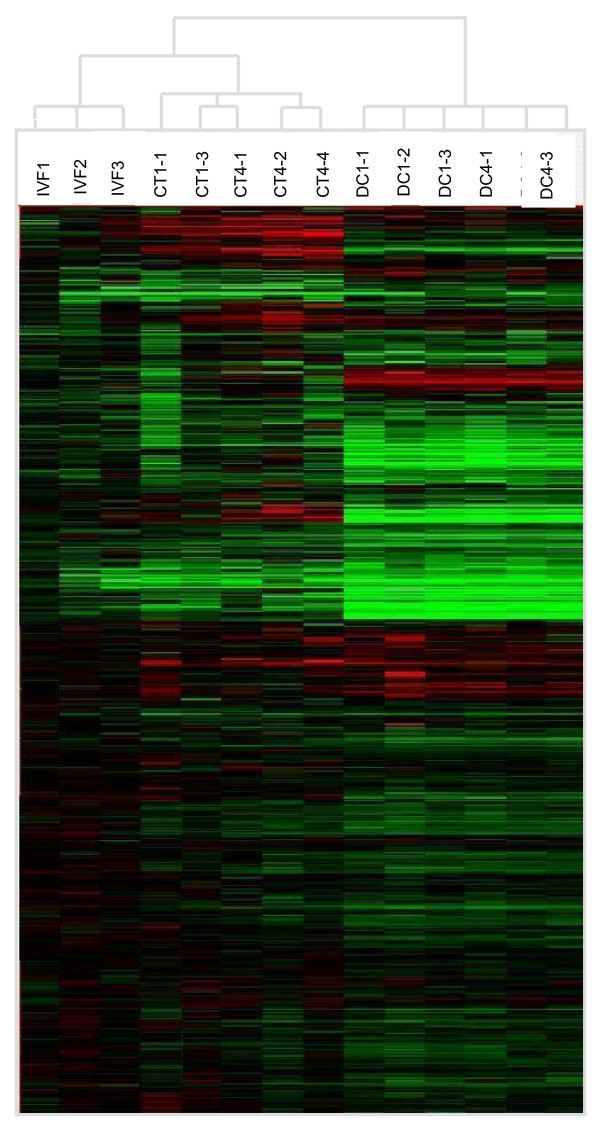
**Hierarchical clustering of microarray hybridizations**. Cluster analysis of hybridizations and genes performed using GeneTraffic UNO (Iobion Informatics LLC). All donor cells were clustered in one group, while all the embryos were clustered in a second group. The embryos clearly separate into two groups: a group containing the IVF embryos and a group containing the chromatin transfer embryos.

Hierarchical clustering classified all donor cells chips in one single group indicating small differences in their gene expression profiles. All blastocysts were classified in 2 distinctive clusters with IVF blastocysts in one group and all cloned blastocysts in other group (Figure 2).

In pairwise comparisons among transcripts with a p-value < 0.01, a False Discovery Rate (FDR) of 20%, and a Fold Change >2.0 were considered differentially expressed. The numbers of differentially expressed transcripts in all pairwise comparisons are presented in Table 1. The number of probe sets that were differentially expressed between all 3 groups of blastocysts was significantly lower compared to the number of differentially expressed transcripts between donor cells and embryos (P < 0.01). This numeric difference indicates that a substantial reprogramming has occurred in cloned blastocysts from both first and fourth rounds of cloning. However there were significantly less differentially expressed transcripts between cloned embryos and donor cells than between IVF blastocysts and donor cells (P < 0.01). Out of 83 differentially expressed transcripts between both cell lines, 79 corresponded to absent or marginal signals, leaving only 4 differentially expressed transcripts. Chemokine binding protein 2 (CCBP2) and myocilin, trabecular meshwork inducible glucocorticoid response (MYOC) were upregulated in DC1 compared to DC4. Similar to hemicentin (LOC528634) and similar to dolichyl pyrophosphate phosphatase 1 (LOC504908) were the genes upregulated in DC4 compared to DC1.

**Table 1:** Number of differentially expressed transcripts in pairwise comparisons between IVF embryos, CT1 embryos, CT4 embryos, DC1 cells, and DC4 cells (p-value < 0.01 and fold change > 2.0)

	**Comparison** **Group 1 *vs*. Group 2**	**Differentially expressed transcripts**	**Higher in the first group**	**Higher in the second group**
1	IVF embryos *vs*. CT-1 embryos	270^a^	123	147
2	IVF embryos *vs*. CT-4 embryos	411^a^	193	218
3	IVF embryos *vs*. DC-1 cells	3360^c^	1548	1812
4	IVF embryos *vs*. DC-4 cells	3428^c^	1593	1835
5	CT-1 embryos *vs*. CT-4 embryos	193^a^	91	101
6	CT-1 embryos *vs*. DC-1 cells	2459^b^	1238	1221
7	CT-1 embryos *vs*. DC-4 cells	2588^b^	1379	1209
8	CT-4 embryos *vs*. DC-1 cells	2036^b^	1151	885
9	CT-4 embryos *vs*. DC-4 cells	2276^b^	1287	989
10	DC-1 cells *vs*. DC-4 cells	83^d^	34	49

Different subscripts indicate statistically significant differences in the number of differentially expressed transcripts.

Because the bovine genome has not been fully annotated, the annotation information available from NetAffx Analysis Center (Affymetrix) classifies probe sets as: 1) fully annotated bovine genes; 2) transcripts similar to specific genes, but not confirmed; 3) hypothetical proteins based on sequence similarity; 4) cDNA clones; and 5) transcripts with strong, moderate or weak similarity to genes from other species. Table 2 presents a breakdown of the differentially expressed transcripts according to these categories. Only transcripts corresponding to annotated bovine genes were included in further analyses.

**Table 2:** Classification of differentially expressed probe sets in pairwise comparisons

	**Comparisons**									
**Probe set category**	**IVF *vs*. CT1**	**IVF *vs*. CT4**	**IVF *vs*. DC1**	**IVF *vs*. DC4**	**CT1 *vs*. CT4**	**CT1 *vs*. DC1**	**CT1 *vs*. DC4**	**CT4 *vs*. DC1**	**CT4 *vs*. DC4**	**DC1 *vs*. DC4**

Genes	63	104	747	763	44	574	563	421	461	23
Similar to...	106	180	1564	1597	81	1071	1132	898	995	34
Hypothetical proteins	4	10	90	102	0	69	80	65	76	6
cDNA clones	0	1	24	28	0	19	16	17	16	0
Transcripts with strong similarity to a known gene	1	3	26	23	0	17	17	19	21	0
Transcripts with moderate similarity to a known gene	2	0	24	27	2	13	16	12	14	2
Transcripts with weak similarity to a known gene	1	0	13	15	1	10	17	8	10	0
Unknown transcripts	93	113	872	873	64	686	747	596	683	18

**Total**	**270**	**411**	**3360**	**3428**	**192**	**2459**	**2588**	**2036**	**2276**	**83**

The probe set categories correspond to NetAffx Bovine GeneChip (Nov. 2007 Annotation).

Multiple comparisons through one-way analysis of variance (ANOVA) using a Least Significant Differences (LSD) test showed a set of 109 genes that were differentially expressed in the cloned embryos and donor cells compared to their IVF counterparts. Out of 109 genes, 67 were upregulated in IVF embryos compared to CT embryos and donor cells (top 30 in Table 3). Forty two genes were upregulated in CT embryos (top 30 in Table 4).

**Table 3:** Top 30 upregulated genes, in IVF blastocysts compared to CT blastocysts sorted by P-value

**Probe set ID**	**Gene Title**	**Gene ID**	**P value**	**Fold change IVF/CT1**	**Fold change IVF/CT4**
Bt.28010.1.S1_at	Peptidase inhibitor 3, skin-derived (SKALP)	PI3	0.000000	5.58	9.12
Bt.21013.1.S1_at	Polo-like kinase 3 (Drosophila)	PLK3	0.000001	3.99	9.09
Bt.28223.1.S1_at	20-beta-hydroxysteroid dehydrogenase-like	MGC127133	0.000009	2.13	1.71
Bt.9525.1.A1_at	Zinc finger protein 183	ZNF183	0.000057	2.13	3.62
Bt.2892.1.S1_at	Fatty acid binding protein 7, brain	FABP7	0.00014	1.22	6.35
Bt.4430.1.S2_at	ATPase, H+ transporting, lysosomal V0 subunit a1	ATP6V0A1	0.00014	1.89	1.93
Bt.5154.1.S1_at	Heat shock 70 kDa protein 1A	HSPA1A	0.0002	4.14	7.17
Bt.15787.1.S1_at	Bcl-2 inhibitor of transcription	BIT1	0.0002	1.49	2.01
Bt.13544.2.S1_a_at	Zinc finger protein 410	ZNF410	0.0003	2.01	1.90
Bt.2005.1.S1_at	LSM1 homolog, U6 small nuclear RNA associated	LSM1	0.0003	1.86	1.57
Bt.16291.1.A1_at	Testis expressed 12	TEX12	0.0004	3.40	3.64
Bt.27854.1.S1_at	Nuclear factor, interleukin 3 regulated	NFIL3	0.0004	1.72	3.08
Bt.13928.2.S1_a_at	Sodium channel modifier 1	SCNM1	0.0004	2.02	4.05
Bt.15334.2.A1_at	Signal transducer and activator of transcription 3	STAT3	0.0005	4.71	16.06
Bt.12506.1.S1_at	Serpin peptidase inhibitor, E member 2	SERPINE2	0.0005	1.52	1.42
Bt.20204.1.S1_at	Sjogren's syndrome/scleroderma autoantigen	SSSCA1	0.0005	1.61	2.93
Bt.20199.1.A1_at	DEAD (Asp-Glu-Ala-Asp) polypeptide 56	DDX56	0.0006	1.45	1.79
Bt.3359.1.S1_at	General transcription factor IIF, polypeptide	GTF2F1	0.0009	1.53	1.98
Bt.2958.1.A1_at	Ubiquitin-conjugating enzyme E2A (RAD6 homolog)	UBE2A	0.001	2.03	3.08
Bt.3002.1.S1_at	BUB3 budding uninhibited by benzimidazoles 3	BUB3	0.001	1.27	1.62
Bt.6087.1.S1_at	Transmembrane 4 superfamily member 1	TM4SF1	0.001	2.29	6.62
Bt.4737.1.S2_s_at	Prion protein	PRNP	0.001	2.20	2.99
Bt.1854.1.S1_at	Intraflagellar transport 20 homolog (Chlamydomonas)	IFT20	0.001	1.65	2.30
Bt.5340.1.S1_s_at	Nucleoside-diphosphate kinase NBR-A	NBR-A	0.002	1.42	1.76
Bt.8.1.S1_at	Keratin 10 (epidermolytic hyperkeratosis)	KRT10	0.002	1.98	3.39
Bt.27095.1.S1_at	Collaborates/cooperates with ARF protein	CARF	0.002	1.53	2.97
Bt.5039.1.S1_at	High mobility group nucleosomal binding domain 3	HMGN3	0.002	1.72	2.96
Bt.27874.1.S1_s_at	Phosphatidylserine receptor	PTDSR	0.002	2.29	2.99
Bt.4595.1.S1_at	TSR2, 20S rRNA accumulation, homolog	TSR2	0.002	2.08	2.59
Bt.1505.1.S1_at	Sin3A-associated protein, 18 kDa	SAP18	0.003	2.24	2.48

Genes were analyzed by one-way ANOVA.

**Table 4:** Top 30 genes upregulated in CT blastocysts and donor cells compared to IVF blastocysts, sorted by P-value

**Probe set ID**	**Gene Title**	**Gene ID**	**P value**	**Fold change CT1/IVF**	**Fold change CT4/IVF**
Bt.8933.1.S1_at	Adaptor-related protein complex 3, sigma 2	AP3S2	0.0001	1.61	2.21
Bt.27382.1.A1_s_at	X-ray repair complementing defective repair in Chinese hamster cells 1	XRCC1	0.0001	2.60	2.54
Bt.22224.1.S1_at	insulin receptor substrate 4	IRS4	0.0002	2.31	2.10
Bt.3220.1.S1_at	Crystallin, lambda 1	CRYL1	0.0003	1.94	1.82
Bt.7805.2.S1_a_at	Nuclear casein kinase and cyclin-dependent kinase substrate 1	NUCKS1	0.0003	3.14	3.47
Bt.29540.1.S1_at	Arginine/serine-rich coiled-coil 1	RSRC1	0.0004	1.57	3.09
Bt.19690.1.A1_at	Paraoxonase 1	PON1	0.0005	1.53	3.74
Bt.20444.1.S1_at	thyroid hormone receptor associated protein 5	THRAP	0.0006	1.60	1.20
Bt.16122.1.S1_at	Sorbitol dehydrogenase	SORD	0.0008	2.59	3.40
Bt.5737.1.S1_at	vacuolar protein sorting 26 homolog A	VPS26	0.0008	2.33	3.38
Bt.4292.1.S1_at	ARP3 actin-related protein 3 homolog (yeast)	ACTR3	0.0008	1.69	1.69
Bt.18230.1.S1_a_at	Nuclear autoantigenic sperm protein	NASP	0.0011	1.95	2.75
Bt.9107.1.S1_a_at	phosphatidylinositol binding clathrin assembly protein	PIBCAP	0.0014	1.83	2.93
Bt.663.1.S1_at	Palladin, cytoskeletal associated protein	PALLD	0.0019	2.88	3.08
Bt.1743.2.S1_a_at	Phenylalanyl-tRNA synthetase 2, mitochondrial	FARS2	0.0021	1.64	2.98
Bt.13205.1.A1_at	Mitochondrial ribosomal protein S35	MRPS35	0.0023	1.44	2.29
Bt.25100.1.A1_at	Cortactin	CTTN	0.0026	1.26	1.58
Bt.783.1.S1_at	Aldehyde oxidase 1	AOX1	0.0029	1.99	3.67
Bt.23608.1.S1_s_at	Keratin 8	KRT8	0.0030	3.99	4.59
Bt.27284.1.S1_at	Eukaryotic translation initiation factor 4H isoform 2	WBSCR1	0.0038	2.21	1.55
Bt.10898.1.S1_at	Tumor differentially expressed 2-like	TDE2L	0.0041	1.98	5.08
Bt.28745.1.S1_at	Coagulation factor II receptor-like 1	F2RL1	0.0045	2.25	1.85
Bt.5267.1.S1_at	Annexin A6	ANXA6	0.0046	1.92	3.79
Bt.355.1.S1_at	Caldesmon 1	CALD1	0.0047	1.71	1.79
Bt.20084.2.S1_at	Casein kinase 1, epsilon	CSNK1E	0.0053	3.90	2.64
Bt.2823.3.S1_a_at	Chromosome 1 open reading frame 35	C1orf35	0.0058	1.45	1.78
Bt.7671.1.S1_at	Interferon induced transmembrane protein 1	IFITM1	0.0069	2.47	2.60
Bt.5319.1.S1_at	Anti-oxidant protein 2 (independent phospholipase A2)	AOP2	0.0071	2.03	3.70
Bt.23263.1.S1_s_at	Heat shock 90 kDa protein 1, beta	HSP90AB1	0.0072	1.30	1.90
Bt.19709.1.S1_at	LAG1 homolog, ceramide synthase 2	LASS2	0.0072	1.25	1.69

### Functional classification of genes

**Figure 3:**
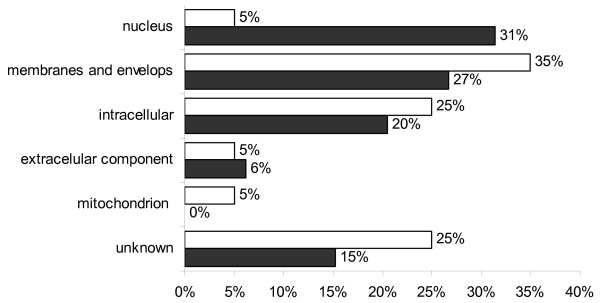
**GoSlimViewer graph of Cellular Component over-represented terms in IVF and CT embryos**. Sub-cellular locations of gene products found at high levels in both IVF blastocysts (solid bars) and both groups of CT blastocysts (open bars). The proportion of genes present in the nucleus was higher in IVF embryos (31%) compared to CT embryos (5%). There were more membrane and intracellular genes in CT embryos compared to IVF embryos.

**Figure 4:**
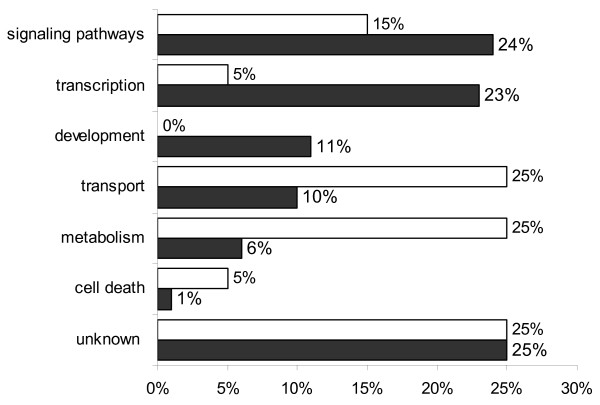
**GoSlimViewer graph of Biological Process over-represented terms in IVF and CT embryos**. Biological processes of gene products found at high levels in both IVF blastocysts (solid bars) and CT blastocysts (open bars). No genes involved in development were upregulated in CT blastocysts compared to IVF blastocysts, for which 11% of the genes were involved in development. Conversely a greater proportion of metabolism genes were overrepresented in CT embryos compared to IVF embryos.

**Figure 5:**
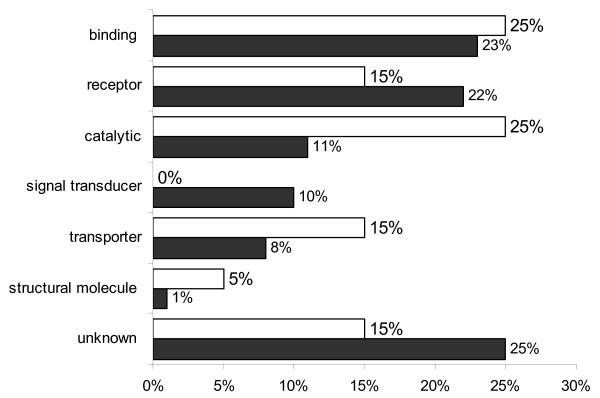
**GoSlimViewer graph of Molecular Function over-represented terms in IVF and CT embryos**. Molecular functions of gene products found at high levels in IVF blastocysts (solid bars) and CT blastocysts (open bars). Genes with receptor function were higher in IVF blastocysts, while genes with catalytic, signal transduction and transporter functions were overrepresented in CT blastocysts.

The Gene Ontology (GO) information for each probe set recovered from NetAffx Analysis Center (Bovine GeneChip November 2007 annotation) was still incomplete for several probe sets, which lacked annotation for at least one of the three ontologies Biological Process (BP), Molecular Function (MF), and Cellular Component (CC). The annotation was complemented with information retrieved using the GOAnna tool part of the AgBase resource at Mississippi State University. All the GO terms associated to each gene were uploaded into the AgBase tool GOSlimViewer in order to obtain a high level summary of the GO categories and create graphs for a better visualization of the data, determining which classes of gene products are over-represented or under-represented on each of the three ontologies for cloned embryos compared to IVF embryos. GOSlimViewer results are summarized in Figures 3, 4 and 5.

### Gene expression analysis by real time RT-PCR

**Figure 6:**
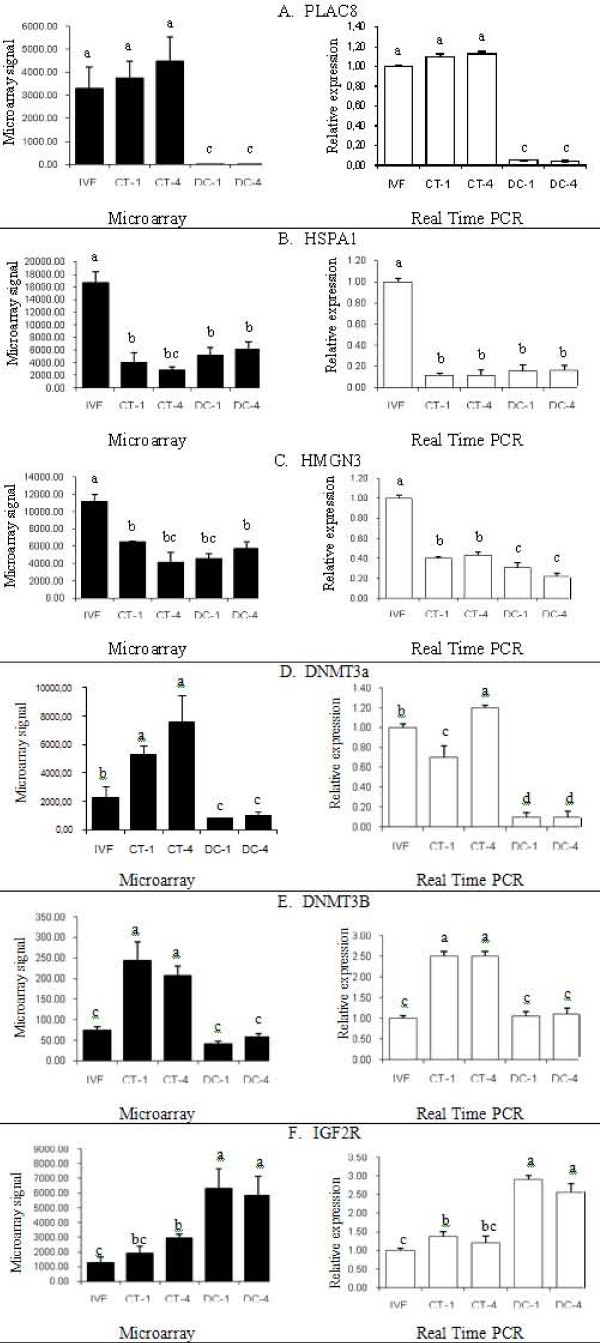
**Real Time PCR gene expression analysis.** Validation of gene expression patterns from the microarray analysis (black bars) by relative quantification through Real time PCR (open bars). A. Validation of gene expression patterns of PLAC8. B. Validation of gene expression patterns of HSPA1. C. Validation of gene expression patterns of HMGN3. D. Validation of gene expression patterns of DNMT3a. E. Validation of gene expression patterns of DNMT3b. F. Validation of gene expression patterns of IGF2R.

**Figure 7:**
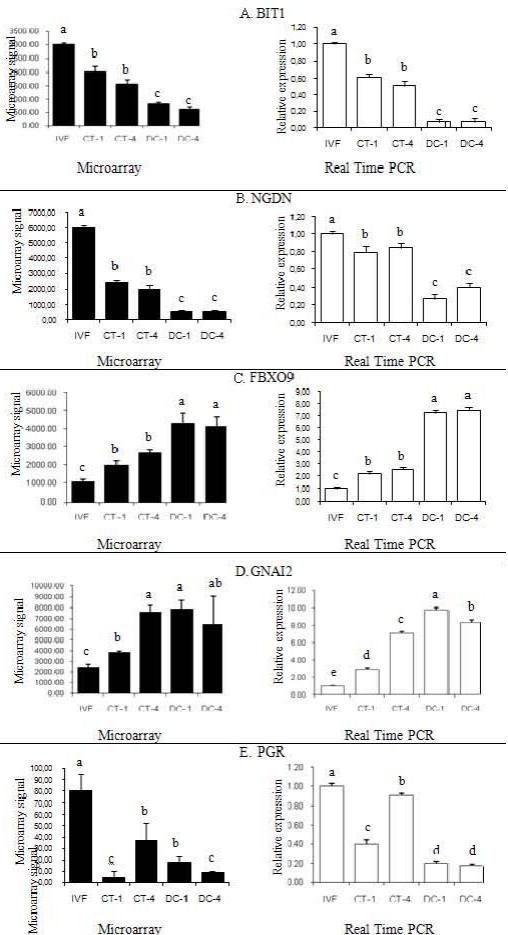
**Real Time PCR gene expression analysis.** Validation of gene expression patterns from the microarray analysis (black bars) by relative quantification through Real time PCR (open bars). A. Validation of gene expression patterns of BIT1. B. Validation of gene expression patterns of NGDN. C. Validation of gene expression patterns of FBXO9. D. Validation of gene expression patterns of GNAI2. E. Validation of gene expression patterns of PGR. Real time PCR units indicate relative expression to the internal standard GAPDH. Different letters on top of each bar indicate significant differences in expression (P < 0.01).

**Figure 8:**
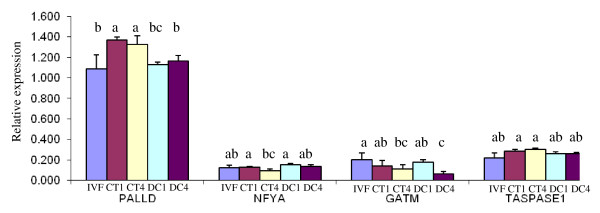
**Real Time PCR gene expression analysis in bovine donor cells.** Gene expression analysis of PALLD, NFYA, GATM and Taspase1 in donor cells lines derived from 0 rounds of cloning (DC0) first round of cloning (DC1), second round of cloning (DC2), fourth round of cloning (DC4), and fifth round of cloning (DC5). Units indicate relative expression to the internal standards GAPDH and 18S rRNA. Different letters indicate significant differences in expression between different donor cell lines (P < 0.01).

In order to confirm the accuracy of microarray data, the following 11 genes were selected based on their relevance during embryonic development: DNMT3A, DNMT3B, IGF2R, PLAC8, PGR, BIT1, HMGN3, HSPA1A, NGDN, FBXO9, and GNAI2 (Table 5). The expression patterns of the selected genes, obtained by Real time PCR, were consistent with the results from the DNA microarray analysis (Figure 6 and 7 ). The analysis of gene expression in the cell lines showed that both housekeeping genes, GAPDH and 18S ribosomal RNA, had a similar pattern of expression. The internal standard 18S ribosomal RNA values were 1.5 times greater in all groups than those of GAPDH. After normalization based on both housekeeping genes, there were no differences among the groups for NFYA and Taspase 1 genes. Both G1 and G2 cell lines had significantly greater concentration of PALLD transcript compared to G0, G4 and, and G5. For GATM, the transcript levels of G5 were significantly lower than in all of the other groups (Figure 8).

**Table 5:** Primers used for Real time PCR validation

**Genes**	**Primer sequences and positions (5' - 3')**	**Fragment size (bp)**	**Accession Number**
GAPDH_F	TGCTGGTGCTGAGTATGTGGT (333–354)	295	[GenBank:XM_865742]
GAPDH_R	AGTCTTCTGGGTGGCAGTGAT (627–648)		
DNMT3A_F	CTGGCTCTTTGAGAATGTGGTG (2372–2394)	236	[GenBank:XM_867643]
DNMT3A_R	TCACTTTGCTGAACTTGGCTATT (2607–2630)		
DNMT3B_F	GGGAAGGAGTTTGGAATAGGAG (698–720)	417	[GenBank:NM_181813]
DNMT3B_R	CTCTGGTTGCTTGTTGTTAGGTT (1114–1137)		
IGF2R_F	AACCAGGTGATTTAGAAAGTGCC (1939–1962)	397	[GenBank:NM_174352]
IGF2R_R	CGCTTCTCGTTATTGTAGGGTG (2335–2357)		
PLAC8_F	TGTTTCACAGCCAGGTTACAGC (168–190)	200	[GenBank:NM_001025325]
PLAC8_R	GGGTCCGATACATTGTCCTCAT (367–389)		
PGR_F	TAAATGACCAGCAAGCAGAAACT (562–585)	394	[GenBank:XM_613908]
PGR_R	GGTAATTGTGCAGCAATAACCTC (955–978)		
BIT1_F	CGGAGCCAGAGGAAGAATGA (75–95)	445	[GenBank:NM_001034519.1]
BIT1_R	TGCTTGTAGGCAGAAACAGCA (519–530)		
HMGN3_F	GTTCCAGCCCGTTGCTTTAC (22–42)	355	[GenBank:NM_001034504.1]
HMGN3_R	GACCATTCATTCTCCCTCGTTAG (376–399)		
HSPA1A_F	CACGATGTTGATCCTGTGGG (86–106)	380	[GenBank:NM_174550.1]
HSPA1A_R	CACCTTAGGCTTGTCTCCGTC (465–487)		
NGDN_F	GTGAGAATGACCCACTCCGTT (403–424)	397	[GenBank:NM_001046459]
NGDN_R	TCCCGCTTGCTGACACTTAA (799–819)		
FBXO9_F	GCAGACGGCAGGAGTAGACAC (231–252)	445	[GenBank:NM_001034412.1]
FBXO9_R	ACAAGTTGCATAGCCCTACGAT (675–697)		
GNAI2_F	TCCAGACAACTGCCAACATCA (1978–1999)	215	[GenBank:XM_589440.3]
GNAI2_R	CAAACCAGGTGAACAATTCCATA (2192–2215)		
PALLD_F	AGGTTGACCTACGAGGAAAGGA (2071–2092)	292	[GenBank:XM_869983.2]
PALLD_R	ATGTGAACGTCGCAGGCATA (2362–2382)		
NFYA_F	CGGGCTAAATTAGAAGCAGAAG (998–1020)	311	[GenBank: NM_001014956.1]
NFYA_R	AGGGCAGAATGTGATCGTCAG (1308–1329)		
GATM_F	ATTGGCTGCTCAGGGAAAGT (824–844)	262	[GenBank: NM_001045878.1]
GATM_R	ACATGGTCGGTCAGGGTTG (1085–1104)		
TASPASE1_F	CAAGACTCATATTTCCAGACTCCC (1145–1169)	264	[GenBank: NM_001034577.1]
TASPASE1-R	CCAAGCACTAACTACAGCAGCAC (1408–1431)		

**Table 6:** Genes with putative cummulative downregulation in blastocysts obtained after serial rounds of chromatin tranfer

**Probe Set ID**	**Gene Title**	**Gene Symbol**	**IVF**	**CT1**	**CT4**	**Fold change IVF/CT1**	**Fold change IVF/CT4**
Bt.5154.1.S1_at	heat shock 70 kD protein 1	HSPA1A	16655.53	4021.00	2975.26	4.14	5.60
Bt.9759.1.S1_a_at	neuroguidin, EIF4E binding protein	NGDN	11691.84	5346.60	3041.70	2.19	3.84
Bt.5039.1.S1_at	high mobility group nucleosomal binding domain 3	HMGN3	11195.32	6522.85	4078.53	1.72	2.74
Bt.9759.2.S1_at	neuroguidin, EIF4E binding protein	NGDN	5999.87	2431.02	1665.35	2.47	3.60
Bt.4737.1.S2_s_at	prion protein	PRNP	3552.73	1614.40	1425.30	2.20	2.49
Bt.1854.1.S1_at	intraflagellar transport protein 20	IFT20	3526.47	2139.25	1380.10	1.65	2.56
Bt.27874.1.S1_s_at	phosphatidylserine receptor	PTDSR	3476.73	1517.25	980.58	2.29	3.55
Bt.15787.1.S1_at	Bcl-2 inhibitor of transcription	BIT1	2989.58	2007.15	1415.27	1.49	2.11
Bt.20204.1.S1_at	Sjogren's syndrome/scleroderma autoantigen 1	SSSCA1	1695.08	1056.05	579.62	1.61	2.92
Bt.4595.1.S1_at	TSR2, 20S rRNA accumulation, homolog (S. cerevisiae)	TSR2	1567.39	755.35	568.11	2.08	2.76
Bt.12250.1.S1_at	chromosome 14 open reading frame 10	C14orf10	1525.13	981.80	567.59	1.55	2.69
Bt.27095.1.S1_at	collaborates/cooperates with ARF (alternate reading frame) protein	CARF	1390.25	907.40	668.85	1.53	2.08
Bt.13928.2.S1_a_at	sodium channel modifier 1	SCNM1	786.05	390.50	249.35	2.01	3.15
Bt.6620.1.S1_at	myosin, heavy polypeptide 7, cardiac muscle, beta	MYH7	673.53	219.15	135.85	3.07	4.96
Bt.19972.1.S1_at	proton-dependent gastrointestinal peptide transporter	PEPT1	567.85	189.46	170.27	3.00	3.34
Bt.28010.1.S1_at	protease inhibitor 3, skin-derived (SKALP)	PI3	510.98	91.50	56.05	5.58	9.12
Bt.5126.1.S1_at	hypertension-related calcium-regulated gene	COMMD5	449.40	335.50	176.24	1.34	2.55
Bt.22523.1.S1_at	dispatched homolog 1 (Drosophila)	DISP1	402.17	174.75	155.13	2.30	2.59
Bt.5828.1.S1_at	SERTA domain containing 1	SERTAD1	357.71	287.95	157.44	1.24	2.27
Bt.333.1.S1_at	transition protein 1 (during histone to protamine replacement)	TNP1	233.38	155.00	98.93	1.51	2.36
Bt.14098.1.S1_at	microtubule-associated protein, RP/EB family, member 2	MAPRE2	199.89	183.45	69.82	1.09	2.86
Bt.4158.1.A1_at	oviduct specific glycoprotein	OVGP1	196.48	168.70	78.09	1.16	2.52
Bt.22856.1.S1_at	neurofilament, medium polypeptide	NEF3	188.69	126.35	46.89	1.49	4.02
Bt.9807.1.S1_at	glycoprotein (transmembrane) nmb	GPNMB	154.95	52.30	24.03	2.96	6.45
Bt.23151.1.S1_at	fucosyltransferase 10 (alpha (1,3) fucosyltransferase)	FUT10	154.43	114.10	55.12	1.35	2.80
Bt.7239.1.S1_at	solute carrier family 6 (neurotransmitter transporter, dopamine), member 3	SLC6A3	149.32	48.30	21.24	3.09	7.03
Bt.12739.2.S1_a_at	membrane-associated ring finger (C3HC4) 2	C3HC4	110.18	51.40	23.87	2.14	4.62
Bt.6556.1.S1_at	regakine-1 protein	LOC504773	89.66	25.75	39.07	3.48	2.29
Bt.12080.2.S1_at	Bernardinelli-Seip congenital lipodystrophy 2	BSCL2	88.59	38.70	13.83	2.29	6.41
Bt.13036.1.S1_at	progesterone receptor	PGR	79.73	4.69	36.57	17.02	2.18
Bt.2157.1.S1_a_at	RPGR-interacting protein 1	RPGRIP1	77.03	58.90	6.56	1.31	11.75
Bt.28409.2.S1_at	DNA replication factor	CDT1	71.69	55.20	12.73	1.30	5.63
Bt.3771.1.A1_at	Nucleolar protein family A, member 1	NOLA1	69.73	21.50	21.26	3.24	3.28
Bt.27752.1.S1_at	tensin 4	TNS4	69.66	43.05	8.73	1.62	7.98
Bt.13024.2.S1_at	purinergic receptor P2Y G-protein coupled, 2	P2RY2	67.08	46.15	22.11	1.45	3.03
Bt.28017.1.S1_at	vacuolar H+-ATPase	LOC407191	65.07	34.20	17.47	1.90	3.73
Bt.512.1.S1_at	nucleotide phosphodiesterase, 3'-5'-cyclic	PDE1A	60.70	15.59	15.85	3.89	3.83
Bt.12928.1.S1_at	Interleukin 13	IL13	58.85	37.70	9.25	1.56	6.36
Bt.29129.1.S1_at	anterior gradient 2 homologue	agr2	45.07	39.00	21.29	1.16	2.12

(P < 0.01).

**Table 7:** Genes with putative cummulative upegulation in blastocysts obtained after serial rounds of chromatin tranfer

**Probe Set ID**	**Gene Title**	**Gene Symbol**	**IVF**	**CT1**	**CT4**	**Fold change IVF/CT1**	**Fold change IVF/CT4**
Bt.4475.1.S1_at	NADH dehydrogenase (ubiquinone) Fe-S protein 2, 49 kDa (NADH-coenzyme Q reductase)	NDUFS2	6724.02	13373.15	14960.42	1.99	2.22
Bt.3583.1.S1_at	villin 2	VIL2	6698.24	13698.40	17209.52	2.05	2.57
Bt.663.1.S1_at	palladin, cytoskeletal associated protein	PALLD	5038.25	14502.45	19368.34	2.88	3.84
Bt.9068.1.S1_at	non-muscle myosin heavy chain	LOC404108	3,972.71	6,504.05	8,152.57	1.64	2.05
Bt.2841.1.S1_at	tryptophanyl-tRNA synthetase	WARS	2,665.06	4,276.85	5,569.16	1.60	2.09
Bt.4311.1.S1_at	guanidine nucleotide binding protein, (G protein), alpha inhibiting activity polypeptide 2	GNAI2	2,389.08	3,859.15	7,740.86	1.62	3.24
Bt.962.1.S1_at	golgi autoantigen, golgin subfamily a, 7	GOLGA7	1,689.70	2,728.90	4,288.07	1.62	2.54
Bt.760.1.S1_at	zinc finger protein 313	Znf313	1,523.55	2,140.45	3,126.63	1.40	2.05
Bt.803.1.A1_at	chromatin modifying protein 1B	CHMP1B	1,315.99	2,093.75	3,934.13	1.59	2.99
Bt.4503.1.S1_at	mitochondrial carrier homolog 2	Mtch2	1,279.84	3,359.75	4,555.63	2.63	3.56
Bt.23603.3.S1_at	F-box protein 9	FBXO9	1,058.76	1,948.25	2,813.78	1.84	2.66
Bt.7169.1.S1_at	methylmalonyl Coenzyme A mutase	MUT	898.23	1,622.10	1,943.02	1.81	2.16
Bt.14010.1.S1_at	leukotriene B4 12-hydroxydehydrogenase	LTB4DH	841.63	5688.55	11345.50	6.76	13.48
Bt.8933.1.S1_at	adaptor-related protein complex 3, sigma 2 subunit	AP3S2	667.54	1,071.50	1,425.67	1.61	2.14
Bt.12261.1.A1_at	taspase 1	C20orf13	435.56	1,113.20	1,293.73	2.56	2.97
Bt.4738.1.S1_at	calpastatin	CAST	329.41	504.45	890.74	1.53	2.70
Bt.26764.1.A1_at	Lectomedin 2	LEC2	307.46	1,085.70	1,567.79	3.53	5.10
Bt.1388.1.S1_at	Abl-philin 2 isoform 2	ZDHHC16	286.19	630.40	948.26	2.20	3.31
Bt.20236.1.S1_at	thrombospondin repeat containing 1	ADAMTSL4	211.65	322.35	522.48	1.52	2.47
Bt.5330.1.S1_at	lysosomal-associated membrane protein 1	LAMP1	194.91	174.90	1,195.48	0.90	6.13
Bt.8870.3.S1_at	CGI-119 protein	CGI-119	128.16	218.35	403.76	1.70	3.15
Bt.23209.1.S1_a_at	lectomedin 2	LEC2	83.06	279.90	418.46	3.37	5.04
Bt.318.1.S1_at	adrenergic, beta 3, receptor	ADRB3	26.17	52.65	89.07	2.01	3.40
Bt.4057.1.S1_at	myosin, heavy polypeptide 10, non-muscle	MYH10	21.07	38.95	83.71	1.85	3.97
Bt.4560.1.S1_s_at	trophoblast Kunitz domain protein 1	TKDP1	21.03	43.25	88.08	2.06	4.19
Bt.22858.1.S1_at	uroplakin IIIB	UPK3B	16.02	16.70	100.71	1.04	6.29
Bt.12304.1.S1_at	interferon-stimulated protein, 15 kDa	ISG15	15.51	67.95	66.46	4.38	4.29
Bt.26830.2.S1_a_at	5,10-methylenetetrahydrofolate reductase (NADPH)	MTHFR	12.02	57.85	79.11	4.81	6.58
Bt.5101.1.S1_at	prion protein interacting protein	PRNPIP	8.97	32.00	74.73	3.57	8.33
Bt.17862.1.A1_at	Guanine nucleotide binding protein (G protein), alpha stimulating activity polypeptide 1	GNAS1	8.03	42.00	44.87	5.23	5.59
Bt.2301.1.S1_at	Zinc finger protein 325 (gonadotropin inducible transcription repressor-3)	ZNF325	3.81	22.10	121.65	5.80	31.93
Bt.17862.1.A1_at	Guanine nucleotide binding protein (G protein), alpha stimulating activity polypeptide 1	GNAS1	8.03	42.00	44.87	5.23	5.59
Bt.12304.1.S1_at	interferon-stimulated protein, 15 kDa	ISG15	15.51	67.95	66.46	4.38	4.29
Bt.12261.1.A1_at	taspase 1	C20orf13	435.56	1113.20	1293.73	2.56	2.97
Bt.3583.1.S1_at	villin 2	VIL2	6698.24	13698.40	17209.52	2.05	2.57

(P < 0.01)

### Data modelling

**Figure 9:**
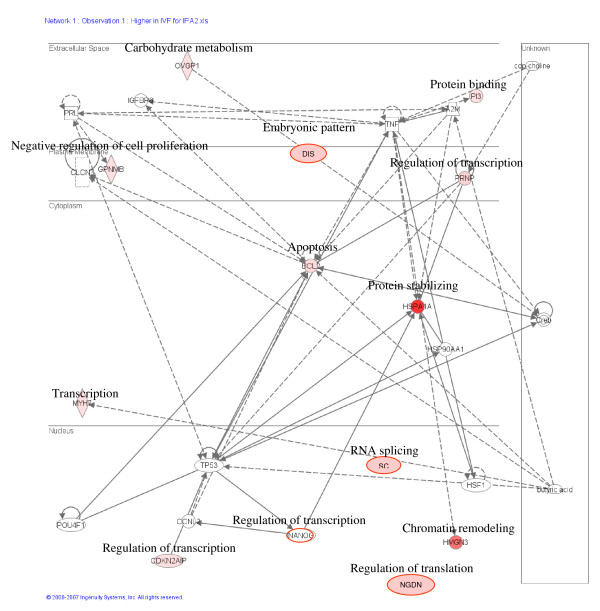
**Display of genes with high expression in IVF embryos.** Data modelling of genes with high expression in IVF embryos compared to cloned embryos. The top networks in the pathway include cellular growth and proliferation, embryonic development, cellular assembly and organization, cellular death and response to stress and cancer.

**Figure 10:**
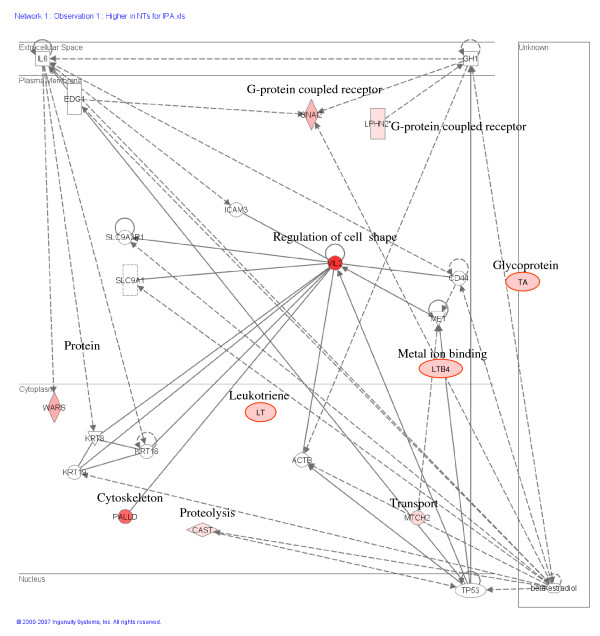
**Display of genes with high expression in CT embryos.** Data modelling of genes with higher expression in CT embryos compared to IVF embryos. The top networks in the pathway include cellular morphology, cellular development, cell signalling and metabolism.

The pathways originated using Ingenuity Pathway Analysis showed the most important pathways in which the differentially expressed genes participate. The top networks formed by the genes upregulated in IVF embryos compared to both CT groups included cellular growth and proliferation, embryonic development, cellular assembly and organization, cellular death and response to stress (Figure 9). On the other hand the networks obtained from the transcripts more abundant in the cloned blastocysts compared to IVF embryos were cellular morphology cellular development, cell signaling, and metabolism (Figure 10). Genes with a putative cumulative misregulation after serial rounds of chromatin transfer are presented on Tables 6 and 7.

## Discussion

It has been reported that in vitro culture conditions alter gene expression and may lead to developmental aberrations in IVF derived cattle, commonly referred to as the large offspring syndrome [38-40]. In the case of embryos produced by SCNT, besides the alterations due to in vitro culture conditions, gene expression defects may be caused by improper silencing and activation of specific genes, altered chromatin remodelling, and epigenetic alterations [41]. But identifying key genes responsible for the general developmental failure in cloned embryos is not an easy task, since the alterations may be caused by a variety of factors including donor cell type, cell cycle stage, nuclear transfer protocol, source of the oocytes, embryo culture system, embryo transfer procedure, recipient cows management, and operators' skills [42]. Consequently, there is a big variety of alterations that are not always shared by all cloned embryos. Still, the common thread uniting many of the SCNT failures can be traced to epigenetic alterations, specifically failures in chromatin remodelling and DNA and histone methylation [13,43,44]. The fetal fibroblast cells used in this study are not fully representative of adult somatic cells. However, these cells were chosen because of their practicality and higher efficiency in SCNT studies.

Microarray analysis has been used to explore the transcriptome profile of cloned embryos relative to that of the donor cells and IVF embryos as a control. However, the appropriate microarray platform is crucial in order to detect changes in particular genes. Smith and colleagues reported similar transcriptome profiles for cloned blastocysts and blastocysts produced by artificial insemination [15]. However, the cDNA microarray used by Smith and colleagues consisted of placenta and spleen cDNA libraries, lacking embryonic genes, which therefore were not analysed. The results from the present study show an extensive reprogramming in cloned embryos by the blastocyst stage. However, the data point to a group of differentially expressed transcripts between IVF and cloned blastocysts.

Serial cloning is often performed for the production of transgenic animals. Although apparently healthy animals can be obtained after serial cloning, the efficiency of cloning decreases from generation to generation despite comparable blastocyst and early pregnancy rates. This increase in pregnancy losses and perinatal deaths could be caused by gene expression defects accumulated throughout the serial cloning procedures, which could be detected in blastocysts, although no phenotypic alterations are observed at this stage. Furthermore, it has been proposed that the extended culture, associated with transfection and selection procedures, may induce changes in the donor cells [36]. The present studies show that serial cloning does not significantly affect transcriptional reprogramming of cloned blastocysts. The global transcriptome profile of blastocysts from four consecutive rounds of cloning did not significantly differ from the one obtained from blastocysts after only one round of cloning. However, for a set of genes, misregulation was significantly greater in the blastocysts obtained from four rounds of cloning (see Tables 6 and 7). However the observed differences between blastocysts from the first and fourth rounds of cloning could be due to the fact that these are different donor cells and not of the same clonal origin.

To our knowledge this is the first study to focus on the influence of serial chromatin transfer on global transcriptome profile of embryos and donor cells. Only a small proportion of the data set generated by the present study corresponded to fully annotated bovine genes (Table 2). The rest of the probe sets were excluded from further analyses due to lack of annotations. Progress in the annotation of the bovine genome will greatly facilitate global gene expression studies in the bovine species.

In the present study, multiple comparisons revealed five distinctive patterns of differential gene expression among all embryos and donor cells. **The first pattern **corresponded to 1,183 transcripts (30.74% of the data set) that had similar abundance in all five groups. Housekeeping genes like GAPD and Actin showed this pattern of expression. **The second pattern **corresponded to genes that had similar expression in IVF and CT embryos, but had a very different pattern of expression in both donor cell lines. We hypothesised that these are genes that switched from the "donor cell gene expression mode" to the "embryo gene expression mode". The majority of the genes in the data set (76.49%) showed this pattern, including some imprinted and embryonic specific genes such as the Oct-4 protein coding gene (POU5F1), which has been reported as differentially expressed for cloned embryos in previous studies [21,23]. Placenta specific 8 (PLAC8) also shows this pattern of expression (Figure 6A). It is possible that some genes, due to their methylation pattern in the somatic cells or to their location in the chromosome, are more likely to be reprogrammed by the oocyte factors.

**The third pattern **corresponded to genes with a similar pattern of expression for CT embryos and donor cells, and a very different expression pattern in IVF embryos. These were 147 (3.81%) genes with apparently incomplete reprogramming, probably with a somatic cell pattern of expression. The heat shock 70 kD protein 1 (HSPA1A), involved in cell protection from stress and apoptosis was significantly higher in IVF embryos when compared to CT embryos and donor cells (Figure 6B). Important embryonic genes showed this pattern of expression. Desmocollin 3 (DSC3) a transmembrane glycoprotein, involved in cell adhesion that belongs to the cadherin family, was present in IVF embryos but was absent in CT embryos and donor cells. The signal transducer and activator of transcription 3 (STAT3), was significantly upregulated in IVF embryos when compared to both groups of cloned embryos and donor cells. A similar pattern was observed for high mobility group nucleosomal binding domain 3 (HMGN3) a gene involved in chromatin remodelling, a vital process during embryonic genome activation (Figure 6C). The importance of both genes during morulae and blastocyst formation could make them good candidates in understanding the lower developmental rates of cloned embryos.

**The fourth **group of genes corresponded to only 85 probe sets (2.21%) with a marked differential expression in all cloned embryos compared to the one observed in both IVF embryos and donor cells. The misregulation of these genes could point to a compensation mechanism after chromatin transfer. Genes with this kind of expression pattern included prostaglandin-endoperoxide synthase (PTGS2) and the transcription factor GATA-2. Both genes had a greater microarray signal in all CT embryos, but low expression in IVF and donor cells. The imprinted gene glycine amidinotransferase (GATM), showed significantly greater values in the cloned embryos compared to IVF embryos and donor cells. Two interesting genes in this group were DNMT3A and DNMT3B transcripts, which are responsible for de novo methylation. Both genes were significantly greater in CT-1 and CT-4 embryos compared to IVF blastocysts (Figure 6D and 6E), which is consistent with the hypermethylation often reported in cloned blastocysts. These results do not agree with previous findings, in which DNMT3A was downregulated in NT embryos compared to IVF embryos [21]. Zhou et al., reported similar levels of DNMT3B for embryos produced *in vivo*, *in vitro*, and by different nuclear transfer methods, including chromatin transfer [9]. These contrasting results confirm that alterations greatly vary and are not shared by all cloned embryos. One limitation of our study is that we have not used in vivo blastocysts which might have provided more biological means and as the physiological standard against in vitro culture conditions.

**A fifth pattern **corresponded to genes that had an increasing or a decreasing pattern of expression from IVF embryos through donor cells showing an intermediate pattern of expression in CT embryos. In total, 245 probe sets showed this pattern of expression with 119 (3.08%) increasing, and 126 (3.28%) decreasing from IVF through DC. It could be assumed that these genes have been partially reprogrammed, since their transcript abundance is in between IVF and donor cells. The imprinted gene insulin-like growth factor 2 receptor (IGF2R), one of the most studied genes in the large offspring syndrome, showed similar expression values in IVF and CT1 embryos, but significantly higher signals in CT4 embryos, and very high signals in both donor cells (Figure 6F). These higher mRNA levels in the fourth generation of cloning could indicate a cumulative misregulation of this gene. The Bcl-2 inhibitor of transcription (BIT1) showed the greatest values in IVF embryos, intermediate values in CT embryos and the lowest values in donor cells (Figure 7A). The nuclear transcription factor Y, alpha (NFYA), showed a similar expression pattern in both IVF and CT1 embryos; although it was significantly lower in CT4 embryos and donor cells. Neuroguidin (NGDN), an eukaryotic translation initiation factor with important functions in embryonic development was another gene with a decreasing pattern of expression (Figure 6B). Genes with and increasing pattern of expression included F-box protein 9 (FBXO9), and guanine nucleotide binding protein alpha inhibiting activity polypeptide 2 (GNAI2) represented in Figure 6C and Figure 6D, respectively. Transcripts for the progesterone receptor (PGR) were significantly higher in IVF embryos compared to CT embryos and donor cells (Figure 6E). Among this group of transcripts could be genes that are cumulatively affected be serial cloning.

Based on the difference in gene expression for RARB, CRAB1, THBS, SERPINB5, and HLA-A, Beyhan et al. suggest a possible role for the retinoic acid signalling pathway in the failures observed in cloned bovine embryos [22]. However the bovine GeneChip does not contain a Retinoic Acid Receptor Beta (RARB) probe set. It only contains a probe set that corresponds to a bovine EST with similarity to the rat RARB (Bt.21044.2.A1_at). In the present data, CRAB1 and THBS2 were slightly higher in IVF embryos, although without statistical significance. They also found differential gene expression among several genes in both donor cells (CDKN1C, COPG2, DCN, GATM, MEST, NDN, NNAT, PON3, and SGCE). In the current study GATM was significantly downregulated in donor cells from the fifth successive generation of chromatin transfer (Figure 7).

At the blastocyst stage there is an extensive reprogramming of cloned embryos leading to very similar transcriptomes in IVF and CT blastocysts. However, there were around 200 differentially expressed genes in both CT embryos compared to IVF. For some genes, the differences were significantly greater in CT4 when compared to CT1, suggesting a possible cumulative missregulation caused by serial cloning. Genes involved in transcription, cellular proliferation, embryonic development, cellular death, and response to stress are over represented in IVF embryos; many of these genes are present in the nucleus, which was the cell component overrepresented in IVF embryos. Genes involved in cell morphology, cell development, and metabolism were over expressed in donor cells and in cloned embryos when compared to IVF, suggesting that they were not properly silenced in the donor nucleus. The up regulation of genes involved in metabolism should be further explored as it could be linked to the large size of cloned animals.

## Conclusion

As gene expression profile can only show one step in cell phenotype and function control, namely transcriptome regulation, proteomic analysis could complement this study by providing a more complete picture of the regulation of embryonic development. With a more complete bovine genome annotation, more of the differentially expressed transcripts could be analyzed further providing more information for the currently unidentified transcripts, which, in the present study represented around 18% of the dataset. Gene Ontology information for a proportion of the differentially expressed genes is still incomplete. Thus, for some of the genes the cellular component is known, but the biological process and/or its molecular function is not documented. It is interesting that the majority of genes upregulated in CT blastocysts participate in metabolism processes, while the percentage of metabolism genes in IVF blastocyst was lower compared to signalling pathway genes.

The present study provides a data set that could be useful in identifying epigenetic errors in cloning and may facilitate our understanding of the reprogramming process in SCCT. Future studies should involve more of the successive generations of cloned embryos and their respective donor cells to identify cumulative misregulated genes. Gene expression studies from fetal, newborn, and placental tissues could identify genes that are responsible for abnormalities, abortions, stillborns and low birth rate. Functional studies should target particular genes that play key roles in molecular reprogramming and early embryo development and manipulate their mRNA concentrations in SCCT embryos, to mimic that of IVF embryos.

## Methods

### *In vitro *fertilization (IVF)

Bovine oocytes were aspirated from 2–8 mm follicles of abattoir-obtained ovaries from Holstein cows and matured in Tissue Culture Medium (TCM-199, Gibco/Invitrogen, Grand Island, NY) supplemented with 0.2 mM pyruvate, 0.5 μg/ml FSH (Sioux Biochemicals, Sioux City, IA), 5 μg/ml LH (Sioux Biochemicals, Sioux City, IA), 10% FCS (Gibco/Invitrogen, Grand Island, NY), 100 U/ml penicillin and 100 μg/ml streptomycin (Gibco/Invitrogen, Grand Island, NY) in 5% CO_2 _in air at 38.5°C. For fertilization, matured oocytes were transferred to fertilization medium and were fertilized using thawed sperm from a Holstein bull separated by Percoll density gradient and further incubated for 24 hours. Presumptive zygotes were transferred to Gardner's culture medium 1 (G1) for 3 days, followed by 3–4 days culture in Gardner's culture medium 2 (G2). Blastocysts were evaluated and graded according the International Embryo Transfer Society (IETS) guidelines [45]. Grade 1 blastocysts were selected, pooled in groups of 3 blastocysts per tube, frozen (with addition of lyses buffer from RNeasy MicroKit (Qiagen Valencia, CA) in liquid nitrogen and stored in -80°C until RNA isolation.

### Chromatin transfer (CT)

*In vitro*-matured oocytes were enucleated at 20 hpm. Bovine fetal fibroblasts after one and four rounds of cloning were trypsinized and washed in Ca/Mg Hank's Balanced Salt Solution (HBSS) and permeabilized by incubation of 50,000 – 100,000 cells in 31.25 units Streptolysin O (SLO-Sigma, St. Louis, MO) in 100 μl for 30 minutes in a 37°C H_2_O bath. Permeabilized fibroblasts were washed, pelleted and incubated in 40 μl of mitotic extract prepared from MDBK cells containing an ATP-generating system (1 mM ATP, 10 mM creatine phosphate and 25 μg/ml creatine kinase) for 30 min at 38°C. At the end of incubation, the reaction mix was diluted with 500 μl of cell culture media (Alpha MEM with 10% FBS), pelleted and resuspended in TL Hepes. These cells were fused to enucleated oocytes, activated 26 h after maturation with 5 μM calcium ionophore for 4 min followed by 10 μg/ml of cycloheximide and 2.5 μg/ml of cytochalasin D for 5 h. After activation, embryos were washed, and cultured in SOF medium for the first 4 days with 8 mg/ml BSA and the last three days with 10% fetal calf serum at 38.5°C and 5% CO_2 _in air. Grade 1 blastocysts were pooled (3 per tube) and frozen, with addition of lysis buffer. Embryos were stored in -80°C until RNA isolation.

### Fourth generation of SCCT embryos

For subsequent rounds of cloning, CT derived bovine blastocysts from the first generation were transferred into hormonally synchronized cows. At seventy-days, pregnancies were interrupted, and foetuses recovered. Fetal fibroblast cultures were established and used for the next chromatin transfer process. The same procedure was done 3 times to provide a fourth generation of clones. Grade 1 blastocysts from the fourth generation were pooled (3 per tube) and frozen, with addition of lysis buffer. Embryos were stored in -80°C until RNA isolation.

### Establishment of fetal fibroblast cell lines

Fetal cell lines were developed according. Seventy-day old male bovine foetuses were recovered and transported to the laboratory in Dulbecco's PBS (DPBS) with 16 ml/ml of antibiotic-antimycotic (Gibco, Grand Island, NY), 4 ml/ml tylosin tartrate (Sigma, St. Louis, MO), and 8 ml/ml fungizone (Gibco). Foetuses were rinsed in DPBS, the head and internal organs were removed, and remaining tissues were finely chopped into pieces with a scalpel blade. The fibroblasts were separated from the tissue pieces using 0.08% trypsin and 0.02% EDTA in PBS (trypsin-EDTA). The cells were seeded onto 100-mm tissue culture plates (Corning, VWR, Chicago, IL) in a minimal essential medium (a-MEM; Gibco) supplemented with 10% fetal calf serum (FCS; Hyclone, Logan, UT), 0.15 g/ml glutamine (Sigma), 0.003% b-mercaptoethanol (Gibco), and antibiotic-antimycotic (Gibco). On the same day of cloning (day 3 of seeding), the cells were harvested using DPBS with trypsin-EDTA solution and were counted. One million cells were frozen in MEM with 10% FCS, dimethyl sulfoxide (Sigma), and lysis buffer.

### RNA Isolation

RNA was isolated from IVF blastocysts, SCCT blastocysts, and donor cells using the RNeasy MicroKit (Qiagen Valencia, CA) according to the manufacturer's specifications. Briefly, embryos and cells frozen at -80°C in lysis buffer were transferred to silica-gel membrane spin columns and washed with RW1 wash buffer and 80% ethanol. Final RNA elution was conducted using 14 μl of RNAse free water provided in the kit. Concentration and purity of isolated RNA were determined using a NanoDrop^® ^ND-1000 Spectrophotometer (NanoDrop Technologies, Wilmington, DE). Integrity and quality were analyzed using a Bioanalyzer 2100 RNA 6000 Picochip kit (Agilent Technologies, Palo Alto, CA).

### Microarray

Microarray hybridizations were performed in triplicate for each of the experimental groups using Affymetrix Bovine DNA Chips as described by the manufacturer (Affymetrix Santa Clara, CA). Briefly, complementary DNA (cDNA) synthesis was performed from 10 ng total RNA using the Two-Cycle cDNA Synthesis Kit (Affymetrix Santa Clara, CA). The MEGAscript^® ^T7 Kit (Ambion, Inc.) was used for the first *in vitro *transcription (IVT). GeneChip IVT Labeling Kit was used for the second IVT and labelling of RNA. Complementary RNA (cRNA) was fragmented and 10 μg of fragmented cRNA were hybridized to the Genechips in a Hybridization Oven, set to 45°C and rotations of 60 rpm for 16 hours. The chips were then washed and stained with streptavidin/phycoerythrin (SAPE) antibody solution using an Affymetrix FS-450 fluidics station. GeneChips were scanned using the Affymetrix GeneChip scanner 3300.

### Microarray data processing

Images were processed with the Affymetrix GeneChip^® ^Operating Software (GCOS) and expression quantified with MAS 5.0, which also provides information on signal, detection and calculated the detection p-value. Signal information is a numeric value indicating transcript abundance for a particular probe set. Detection information indicates whether the transcript is detected (P, present), undetected (A, absent), or if it is at the limit of detection (M, marginal). Detection p-value indicates the significance of the detection call for a probe set. Only probe sets that were called Present in at least one of the five groups were included in the analysis. A total of 5,599 probe sets were excluded from the analysis as they were called Absent in all groups. The data set for further analysis included 18,396 probe sets.

### Hybridization quality check

Metrics like noise, background, Scale factor, and the ratio of intensities of 3' probes to 5' probes for Actin and GAPDH genes were analyzed for chip quality control. Information about the intensities of the spiked in controls (*B. subtilis *genes *lys, phe, thr*, and *dap*), which were mixed with the total RNA at known concentrations at the beginning of the experiment was used to monitor the linear amplification and labelling process independently from the target samples. The performance of the hybridization control genes (*E. coli *genes *BioB, BioC *and *BioD *and P1 Bacteriophage *cre*) was also used for determining the quality of each chip.

### Microarray data analysis

For data visualization, the raw GeneChip signals were uploaded into GeneTraffic UNO (Iobion Informatics LLC), which generated scatter plots of pairwise hybridization comparisons and Heat maps from all hybridizations using hierarchical clustering. Power Atlas, a web-based resource from the University of Alabama at Birmingham, was used to estimating the power of the hybridization given the sample size [46]. HDBStat was used for statistical analysis[47]. Data were quantile-quantile normalized and examined for outliers using Person's correlation. Quality control statistics included a deleted residuals approach [37,47,48]. False discovery rates (FDR) for the genes were calculated using t-test [49]. Fold changes were calculated based upon the unadjusted data means in pairwise comparisons. Probe sets in each pairwise comparison with a p < 0.01, and FDR of <20%, and a Fold Change (FC) in excess of 2.0 were considered to be significant and examined further. For multiple comparisons, One-way analysis of variance (ANOVA) from PROC GLM in SAS 9.1 (SAS Institute inc. Carey, NC) was performed on the complete data set. The Least Significant Difference (LSD) test was used to detect significant differences between groups.

### Annotation

The probe sets corresponding to differentially expressed genes were uploaded into the Affymetrix Netaffx Analysis Center (Bovine GeneChip annotation from November 6 2007) to retrieve updated information regarding gene symbol, gene title, Biological Process (BP), Molecular function (MF), and Cellular Component (CC) [50]. To complement the annotation from Netaffx, we used the GOAnna tool [51] from AgBase, a Mississippi State University curated, web-accessible resource for functional analysis of agricultural plant and animal gene products. For data visualization, all the GO terms associated to each gene were uploaded into GOSlimViewer [52] another AgBase tool that provides a high level summary of the GO categories found in the dataset allowing a better visualization of the data.

### Data modelling

Ingenuity Pathway Analysis 5.0 from Ingenuity Systems was used for data modelling and the analysis of networks related to the generated data sets. Genes upregulated in IVF embryos compared to CT embryos and donor cells (Figure 8) and genes downregulated in IVF embryos compared to CT embryos and donor cells (Figure 9) were uploaded in the Ingenuity Pathway Analysis 5.0. Since Ingenuity Pathway Analysis database is based on human, mouse, and rat genes, some of the bovine names were not recognized by the software, mostly because of different gene symbols. For those genes, we manually identified the human orthologous symbol.

### Real time RT-PCR gene expression analysis

DNA microarray derived gene expression results for genes DNMT3A, DNMT3B, IGF2R, PLAC8, PGR, BIT1, HMGN3, HSPA1A, NGDN, FBXO9, and GNAI2 were confirmed by Real time PCR using GAPDH as the reference gene. Complementary DNA was generated with the First-Strand cDNA Synthesis system for RT-PCR using SuperScript III Platinum^® ^Two-Step qRT-PCR Kit (Invitrogen Life Technologies, Carlsbad, CA) according to the manufacturer's protocol. The samples were incubated for 10 min at 25°C, 50 min at 42°C and at 85°C for 5 min. Then 2 U of E. coli Rnase H was added to each tube and incubated at 37°C for 20 min. The cDNA was used for quantitative real-time PCR amplification with SYBR Green I chemistry (Roche Applied Sciences, Indianapolis, IN). Real-time quantitative PCR was performed using the LightCyclerTM instrument (Roche Applied Sciences, Indianapolis, IN). The real time PCR reactions were carried out in a total volume of 10 μl according to the manufacturer's manuals for DNA Master SYBR Green I mix (Roche Applied Sciences, IN). The primer concentrations were adjusted to 0.5 μM for each gene. Primers were designed using Primer Premier 5 software (Premier Biosoft International, Palo Alto, CA). Primer sequences used for real time PCR are shown in Table 1. The cycling parameters were 30 seconds at 95°C for denaturation, 50 cycles of 2 seconds at 95°C, 10 seconds at 55°C for amplification (quantification was performed at this step), and 12 seconds at 72°C for extension. The specificity of all individual amplification reactions was confirmed by melting curve analysis. Real-time expression values were calculated through the relative standard curve method, using 10-fold serial dilutions for both the target and the endogenous reference genes by measuring the cycle number at which exponential amplification occurred in a dilution series of samples. Values were normalized to the relative amounts of the control mRNA, which were obtained from a similar standard curve. In real time PCR reactions, the same initial amounts of target molecules were used, and the Cp values of control mRNA were constant in all samples.

### Real time RT-PCR gene expression analysis from bovine fetal donor cells

Donor cell lines included in the study were fibroblasts from non-cloned foetuses (DC0), and fetal fibroblasts from first, second, fourth, and fifth rounds of cloning (DC1, DC2, DC4, and DC5). RNA isolation from donor cells and subsequent cDNA synthesis were performed according to the above mentioned protocols. Relative mRNA abundance was determined for paladin (PALLD), nuclear transcription factor Y alpha (NFYA), glycine amidinotransferase (GATM) and Taspase 1 (C20orf13). Quantitative assessment of RNA amplification was detected by SYBR^® ^GreenER™ qPCR SuperMixes for iCycler (Invitrogen Life Technologies, Carlsbad, CA, 11761-100). Real-time PCR reactions were performed using the iCycler iQ Real-Time PCR instrument (BIO-RAD). The cycling parameters were 50°C for 2 min, 95°C for 8 min 30 s for denaturation, 40 cycles of 15 s at 95°C and 30 s at 60°C and 30 s at 72°C for amplification and extension respectively. The melting curve was performed starting at 55°C with a 0.5°C increase for 10 s in 80 cycles. Expression values were calculated using the relative standard curve method. Standard curves were generated using 10-fold serial dilutions for both GAPDH and 18S ribosomal RNA. Standard curves were also generated for all target genes by measuring the cycle number at which exponential amplification occurred.

### Statistical analysis of Real Time PCR results

Results from different groups were analyzed by one-way analysis of variance (ANOVA) by SAS 9.1 (SAS Institute inc. Carey, NC). Differences at p < 0.001 were considered significant. An additional analysis was performed using Relative expression software tool (REST©, 384-beta version May 2005) to compare all samples of each group. The mathematical model used in the REST software is based on the PCR efficiencies (E) and the crossing point deviation between the samples (CP) [53-55].

## Abbreviations

BSA: Bovine Serum Albumin; cDNA: complementary DNA; cRNA: Complementary RNA; CT: Chromatin transfer; DC: Donor cell; FCS: Fetal Calf Serum; GO: Gene Ontology; IVF: *in vitro *fertilization; mRNA: messenger RNA; SCNT: Somatic cell nuclear transfer; SCCT: Somatic cell chromatin transfer; CT: chromatin transfer.

## Authors' contributions

NRO performed RNA isolations, microarray experiments, microarray analysis, Real Time PCR experiments and analysis and drafted the manuscript. ZW participated in the design of the study, in its coordination and helped to draft the manuscript. PK carried out the somatic cell chromatin transfers and the *in vitro *fertilizations, participated in the design of the study and helped to draft the manuscript. GPP performed the statistical analysis of microarray data. JMR participated in the design of the study and helped to draft the manuscript. EM participated in the design of the study, in its coordination and helped to draft the manuscript.
